# Desmoplastic Melanoma: Clinical Behavior and Management Implications

**Published:** 2016-01-11

**Authors:** Collier S. Pace, Jyoti P. Kapil, Luke G. Wolfe, Brian J. Kaplan, James P. Neifeld

**Affiliations:** ^a^Division of Plastic and Reconstructive Surgery, Department of Surgery, Virginia Commonwealth University Medical Center, Richmond; ^b^Department of Pathology, Virginia Commonwealth University Medical Center, Richmond; ^c^Division of Surgical Oncology, Department of Surgery, Massey Cancer Center, Virginia Commonwealth University Medical Center, Richmond

**Keywords:** desmoplastic melanoma, local recurrence, regional metastasis, pure desmoplastic melanoma, skin cancer

## Abstract

**Introduction:** Desmoplastic melanoma is a rare variant of melanoma that has been reported to demonstrate unique clinical behavior when compared with other histological subtypes. In this study, we present the clinical course of patients with this unusual diagnosis. We hypothesized that desmoplastic melanoma would differ from nondesmoplastic melanoma with regard to its presentation, rate of regional metastasis, and recurrence pattern. **Methods:** After institutional review board approval, a retrospective chart review was performed on all patients with a diagnosis of desmoplastic melanoma since 1998. The following data were collected: patient demographics, histopathological details of the lesion, initial treatment, and clinical course. In addition, the available slides were reviewed by a dermatopathologist. **Results:** Twenty-eight patient charts were reviewed. Mean age at diagnosis was 65 years. Fifty-seven percent of patients were men, and 67% of the lesions originated from the head and neck. Of the 28 patients, 11 had pathology slides that were adequate for evaluation. Pure desmoplastic melanoma, defined by more than 90% of the specimen demonstrating desmoplastic features, was found in only 3 patients. Taking into account all cases, the mean Breslow thickness was 5.09 mm and ulceration was present in 12.5% of lesions. Regional disease was discovered in 18% of patients. The mean follow-up time was 43 months, and the overall recurrence rate was 32%. 66.7% of first recurrences were local. Two of 3 patients with pure desmoplastic melanoma developed regional metastasis. **Conclusions:** Our data largely support previous studies that suggest desmoplastic melanoma behaves differently compared with other histological subtypes. However, the incidence of regional disease among patients with pure desmoplastic melanoma appears to be higher in our study than in previous reports. Although this rare variant typically presents with advanced local disease, the rate of regional metastasis is less than what would be expected for similar thickness, nondesmoplastic cutaneous melanoma. The recurrence pattern is different compared with nondesmoplastic melanoma, and the most common site of recurrence is local. Discrepancy in the literature regarding the clinical behavior of this disease may be related to inconsistent pathological criteria for diagnosis. Further research will help clarify the optimal management of desmoplastic melanoma.

Desmoplastic melanoma (DM) is a rare histological variant of melanoma that may have a distinct clinical behavior when compared with other subtypes. It was first described in 1971 by Conley et al^1^ as having marked spindle cell proliferation and prominent fibrotic stroma. Previous reports have suggested that approximately 1% of cutaneous melanoma is desmoplastic.^2^ At least partially due to the rarity of this disease, there is some discrepancy in the literature regarding its natural history. Some authors feel that DM has a higher propensity for local recurrence and is less likely to metastasize to regional lymph nodes than do the other subtypes.^3^ Others have found the recurrence pattern of DM to be less unique. More recent literature has delineated pure DM, which has desmoplastic features in at least 90% of the specimen, from partial DM (<90%).^4^ These studies show that the unique recurrence pattern and behavior are more pronounced for lesions with a higher proportion of desmoplastic features (Fig 1).^5^^,^^6^.

The purpose of this study was to examine our series of patients with this rare diagnosis and report their clinical course. We hypothesized that DM would differ from non-DM with regard to its clinical presentation, rate of regional metastasis, and recurrence pattern.

## METHODS

Approval from the institutional review board was obtained. Our Department of Pathology provided a list of names and medical record numbers for all patients with a pathological diagnosis of DM from 1998 to 2012. A retrospective chart review was performed on 28 patients with pathology reports describing cutaneous DM who were treated within our Division of Surgical Oncology.

The following demographic data were obtained: date of birth, sex, date of diagnosis, and ethnicity. Data for sun exposure, body mass index, and smoking history were inconsistently available in the charts and therefore were not analyzed.

The following data were recorded regarding the lesion: primary site, diagnostic procedure, Breslow thickness, Clark level, ulceration, perineural involvement, S-100 staining, mitotic figures per high-powered field (hpf), presence of tumor-infiltrating lymphocytes, American Joint Committee on Cancer stage, date of definitive surgical procedure, surgical margins, final pathological margins, clinical lymph node status, lymph node procedure performed, total lymph nodes excised, and total lymph nodes positive. Additional histochemical studies, such as HMB 45, were inconsistently performed and therefore not analyzed.

**Figure 2:**
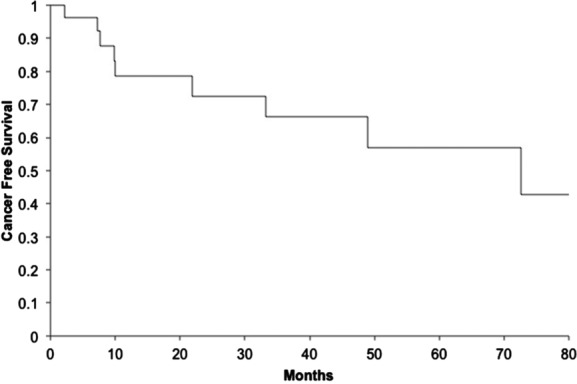
Disease-free survival. Kaplan-Meier curve for disease-free survival.

The date, location, and treatment of each recurrence were recorded. In addition, the most recent date of follow-up was documented. Mortality data were not reliably available in our system and therefore not analyzed. Cancer-free survival was calculated by the Kaplan-Meier method (Fig 2).

To assess the pattern of recurrence, the following variables were correlated with recurrence: age at diagnosis, Breslow thickness, Clark level, mitotic figures per hpf, surgical margins, pathological margins, ulceration, and perineural involvement. For continuous variables, Wilcoxon rank tests were performed. For categorical variables, the Fisher exact test was used.

To clarify the degree of desmoplasia in each specimen, a dermatopathologist (J.P.K.) reviewed the slides that were available. Eleven patients’ slides were reviewed and classified as either pure DM or partial DM. Lesions with desmoplastic features (paucicellular atypical spindled melanocytes, dermal fibrosis, lymphocytic aggregates) involving at least 90% of the specimen were classified as pure.

## RESULTS

Our demographic data (Table 1) are similar to those of many previous studies in showing a slight male predominance. The most common primary site was the head and neck. The mean age at diagnosis was 65 years, and all patients were Caucasian.

The patients in this series presented with relatively advanced local disease (Table 2). Mean Breslow thickness was 5.09 mm, and all lesions were at least a Clark level 4. Ulceration was present in 12.5% of cases, and 30% of lesions examined showed perineural invasion.

The rate of regional metastasis was less than expected for a group of patients presenting with such locally advanced cutaneous melanoma. Only 5 patients (18%) had evidence of regional metastasis at some point in their treatment course. A total of 9 patients (32%) underwent sentinel lymph node biopsy (SLNB), one of which yielded a positive result (see Table 3). Indications for SLNB varied over the course of the study. Patients with lesions of the extremity or trunk were much more likely to receive an SLNB (78%) than patients with lesions of the head and neck (11%). Regional lymph node dissection was performed in 6 patients (21%). One patient received a lymph node dissection after positive SLNB, 1 patient for clinical lymphadenopathy, 1 patient for a clinically concerning lesion superficial to the parotid (the superficial parotid nodes were negative for tumor), and 3 patients for regional recurrence. The patient presenting with clinical lymphadenopathy had a lesion on the cheek (Breslow thickness 6.4 mm) with involvement of the parotid nodes. Wide local excision with parotid and cervical lymph node dissections yielded 6 of 71 lymph nodes with metastasis. The patient developed pulmonary metastases 1 year later. Of patients who developed regional metastasis, all but one of them had lesions of the head and neck. The mean Breslow thickness was 5.37 mm. Two patients were classified as having pure DM, whereas the remaining 3 patients did not have slides acceptable for histological review.

Of the 9 patients (32%) in this series who developed a recurrence, local recurrence was the most common (see Table 4). The initial site of recurrence was local in 6 patients, regional in 2 patients, and distant in 1 patient. Four patients underwent adjuvant radiation therapy as part of their initial treatment due to particularly aggressive local disease or a positive margin on initial wide local excision. None of these patients developed local recurrence. Over the course of our review, 5 patients developed distant metastases. The site of distant metastasis was different in each patient and occurred in the following locations: lung, liver/brain, retroperitoneum, leg and hip. Mean follow-up time was 43 months.

None of the variables evaluated in this study showed a significant correlation with recurrence. This may be due to the small sample size. Neither surgical nor pathological margins were available with enough consistency to allow reliable conclusions.

Of the 11 specimens that were felt to be of acceptable quality for histological review, 3 were classified as pure DM. The remaining 8 were considered partial DM.

## DISCUSSION

Desmoplastic melanoma is a rare variant of cutaneous melanoma that displays a unique clinical course compared with other subtypes. It has been suggested that DM behaves more like a soft-tissue sarcoma than cutaneous melanoma.^3^ The data from our series support previous reports in that patients presented with locally advanced disease, the rate of regional metastasis was relatively low, and recurrence most commonly occurred locally.^7^ The rare nature of this tumor makes it difficult to accumulate enough patients to draw statistically significant conclusions that influence management. Therefore, it is important to share clinical experience with DM and add to the cumulative understanding of this disease.

Some of the discrepancy regarding the behavior of DM and outcomes may be attributed to inconsistent histological characterization. Arbiser et al^8^ described DM as a challenging lesion to diagnose, and second opinions are sought relatively frequently for clarification. In addition, the reports of differences in the clinical behavior of pure DM versus partial DM by Maurichi et al^5^ introduce a confounding variable into all studies of the disease that do not take this into account. They report the incidence of regional disease at presentation, incidence of local recurrence, and incidence of regional recurrence to be 14%, 18%, and 4%, respectively, for partial DM, as compared with 3%, 40%, and 2%, respectively, for pure DM. They suggested a statistically significant increased incidence of local recurrence and decreased propensity for regional spread for pure DM.^5^ Unfortunately, in our series, only 11 of 28 patients had slides of sufficient quality available for evaluation. A total of 3 patients in our series had confirmed pure DM. It is interesting to note that of these 3 patients, 2 developed regional disease. This is contradictory to the belief that pure DM has a lower incidence of regional metastasis. The correlation between the degree of desmoplasia and the unique clinical behavior of DM is a fascinating concept, and it will be interesting to see how this idea will influence management in the future.

The patients with pure DM seem to have pathological characteristics that are otherwise consistent with the remainder of the series, so it is difficult to explain the higher incidence of regional metastases within this subset. The first patient presented at 49 years of age with a scalp lesion. The lesion was 6.2 mm in depth, Clark level 5, no ulceration, positive perineural involvement, and 1 mitotic figure per hpf. The lesion was widely excised with negative margins and recurred 4 years later in the cervical lymph nodes. Lymph node dissection revealed 1 positive node of the 35 examined. A year later, the disease recurred again with local involvement as well as distant bony metastases to the hip. The second patient presented at 69 years of age with an upper extremity lesion. The lesion was 1.44 mm in depth, Clark level 4, no ulceration or perineural involvement, and zero mitotic figure per hpf. The lesion was widely excised with negative margins and an SLNB was performed, which was negative. This patient went on to do well with no recurrence. The third patient presented at 44 years of age with a facial lesion. The lesion was 2.39 mm in depth, Clark level 4, no ulceration, positive perineural involvement, and zero mitotic figure per hpf. The lesion was widely excised with negative margins and showed evidence of local and regional recurrences 7 months later. A reexcision and lymph node dissection was performed revealing 8 of 38 cervical lymph nodes positive for tumor. He went on to develop retroperitoneal metastasis after further surgery and radiation therapy. With such a small number of patients with confirmed pure DM, it is difficult to draw conclusions. However, the incidence of regional disease in this subset of patients contradicts previous studies. We acknowledge there are confounding influences on these data as less than half of the lesions in our study had slides available to allow classification of pure and partial DM. However, even if every one of the lesions that were not able to be classified were classified as pure DM, the incidence of regional metastasis would still be much higher than that reported by Maurichi et al^5^ and Hawkins et al^6^ (1%–1.7%).

The locally aggressive nature of DM is a common theme in the literature. In our series, the lesions had a mean Breslow thickness of 5.09 mm and a 21% incidence of local recurrence. The incidence of local recurrence for DM reported in the literature has ranged from 11% to 40%.^3^^,^^7^ Pasquali et al^9^ reported a local recurrence rate of 7.6% for all subtypes of cutaneous melanoma greater than 4.0 mm in thickness, which is markedly less than what is typically seen in patients with DM. The consistently high incidence of local recurrence seen with DM calls current local management into question. The randomized trials that our current standards for surgical margins are derived from contain a paucity of patients with DM. It may be that desmoplastic lesions, especially pure DM, warrant wider margins at initial excision. Quinn et al^7^ reported a decreased incidence of local recurrence when a minimum of 2-cm margins were achieved, and Maurichi et al^5^ demonstrated improved outcomes regarding survival and recurrence when greater than 2-cm margins were taken for lesions less than 2 mm in thickness. These data suggest that a minimum surgical margin of 2 cm should be obtained in all pure DM lesions when possible, even when the lesion is relatively thin.

Radiation therapy is another way to decrease local recurrence. Historically melanoma was thought to be relatively resistant to radiation. For cutaneous melanoma as a whole, radiation therapy may be used in the palliative or adjuvant setting. Several studies have reported an improvement in locoregional control with adjuvant radiotherapy.^10^^,^^11^ With regard to DM, a recent retrospective study authored by Guadagnolo et al^12^ reports a decreased local recurrence rate in patients who received adjuvant radiotherapy (7%) compared with those who did not (24%). In our study, none of the 4 patients who received adjuvant radiotherapy as part of their initial treatment developed a local recurrence. This is especially noteworthy, as these patients received adjuvant radiation therapy due to features of their initial lesion that would predispose to local recurrence. Prospective analyses will help clarify the role of radiation therapy for DM.

Another area of controversy regarding DM is management of regional lymph nodes. Similar to resection margins, the studies that have guided our criteria for SLNB in cutaneous melanoma group all subtypes together. If DM displays a decreased propensity to spread to regional lymph nodes compared with cutaneous melanoma overall, then criteria for SLNB for DM may differ. In our series, 18% of patients developed regional spread of disease at some point in their treatment course and 1 of the 9 SLNBs performed was positive (11%). When taking the Breslow thickness of our series into consideration, this is much lower than would be expected for typical cutaneous melanoma. Studies examining SLNB for thick cutaneous melanoma (>4 mm) report microscopically detected metastases in 30% to 53% of patients.^13^^-^16 Such discrepancies in the natural history between DM and cutaneous melanoma raise questions as to whether current management guidelines for patients with “typical” melanoma should apply to patients with DM. However, until there is a consensus on the most effective way to manage this aspect of DM treatment, SLNB should be performed according to current standards for cutaneous melanoma.

In conclusion, our series of patients had characteristics and outcomes that are, for the most part, consistent with previous publications. They demonstrated a high incidence of local recurrence, a relatively low incidence of regional metastasis, and presented with locally aggressive disease. Future research with a larger series should examine finer pathological details such as the stratification of the degree of desmoplasia in the specimen to see how that might influence natural history and subsequent management. While many studies suggest that pure DM has a lower than expected incidence of regional metastasis, our data show that 2 of 3 patients with pure DM developed regional disease. In addition, the local management strategy for DM should be reexamined, given the consistently high rates of local recurrence. Collaboration between centers may help achieve the number of patients necessary to aid in our understanding of this rare tumor

## Figures and Tables

**Figure 1:**
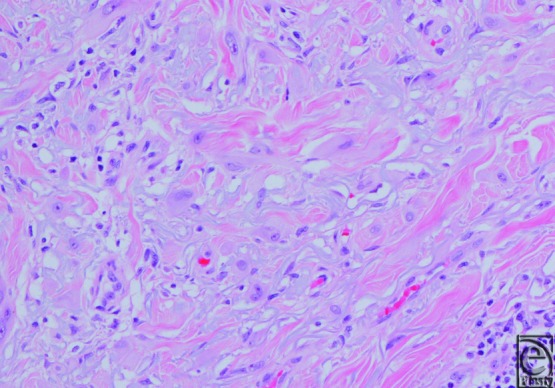
Pure DM with paucicellular atypical spindled cells embedded in a fibrotic stroma.

**Table 1:** Demographics

	Number of Patients	% Total
Sex		
Female	12	43
Male	16	57
Race		
Caucasian	28	100
Age at diagnosis, y		
30–49	5	18
50–69	8	29
≥70	15	53

**Table 2:** Characteristics of primary lesion and stage*

	Number of patients	% Total
Location (*n* = 28)		
Scalp	9	32.1
Face	6	21.4
Neck	4	14.3
Trunk	4	14.3
Leg	3	10.7
Arm	2	7.2
Breslow thickness, mm (*n* = 26)		
≤1.00	0	0
1.01–2.00	9	34.6
2.01–4.00	4	15.4
>4.00	13	50.0
Clark level (*n* = 26)		
1–3	0	0
4	15	57.7
5	11	42.3
Mitoses (*n* = 23)		
<1/hpf	5	21.7
1–4/hpf	10	43.5
>4/hpf	8	34.8
Perineural involvement (*n* = 23)	7	30.4
Ulceration (*n* = 24)	3	12.5
AJCC stage (*n* = 26)		
1a	0	0
1b	8	30.8
2a	5	19.2
2b	7	27.0
2c	3	11.6
3a	1	3.8
3b	0	0
3c	1	3.8
4	1	3.8

*AJCC indicates American Joint Committee on Cancer; hpf, high-powered field.

**Table 3:** Initial management*

	Number of patients	% Total
Initial surgical procedure (*n* = 26)		
WLE	15	57.7
WLE + SLNB	9	34.6
WLE + LND	2	7.7
Final margin status (*n* = 28)		
Negative	20	71.4
Positive or unknown	8	28.6
SLNB status (*n* = 9)		
Negative	8	88.9
Positive	1	11.1
Adjuvant therapy (*n* = 28)		
None	24	85.7
XRT	3	10.7
XRT + chemotherapy	1	3.6

*WLE indicates wide local excision; SLNB, sentinel lymph node biopsy; LND, lymph node dissection; and XRT, radiation therapy.

**Table 4:** Recurrence

	Number of patients	% Total
Overall recurrence (*n* = 28)		
Yes	9	32.1
No	19	67.9
Location of first recurrence (*n* = 9)		
Local	6	66.7
Regional	2	22.2
Distant	1	11.1
Time to first recurrence, y (*n* = 9)		
<1	5	55.6
1–5	3	33.3
>5	1	11.1
